# A Common Language for the Multidisciplinary Team: Point‐of‐Care Musculoskeletal Ultrasound in Modern Haemophilia Care

**DOI:** 10.1111/hae.70411

**Published:** 2026-09-20

**Authors:** Hortensia De la Corte‐Rodriguez, María Teresa Álvarez‐Román, Mauro Davoli, Magnus Aspdahl, Christina Burgess, Karin Fijinvandraat, Roberta Gualtierotti, Cedric Hermans, Marko Marinić, Carlo Martinoli, Wolfgang Miesbach, Gianluigi Pasta, Michael Sigl‐Kraetzig, David Stephensen, Malin Svennefalk, Víctor Jiménez‐Yuste, the E.A.H.A.D. Joint Health Group

**Affiliations:** ^1^ Department of Physical Medicine and Rehabilitation La Paz University Hospital Madrid Spain; ^2^ IdiPAZ Institute for Health Research Madrid Spain; ^3^ University Alfonso X El Sabio Madrid Spain; ^4^ Hematology Department Hospital Universitario La Paz Madrid Spain; ^5^ Medical Director Hemophilia Foundation of the Province of Santa Fe Rosario Argentina; ^6^ Argentine Association of Hemophilia and Associated Disorders (AAHDA) Rosario Argentina; ^7^ Medical Unit Allied Health Professionals Karolinska University Hospital Stockholm Sweden; ^8^ HBDCA (Haemophilia and Bleeding Disorders Counselling Association) Ely UK; ^9^ Amsterdam UMC Pediatric Hematology University of Amsterdam Emma Children's Hospital Amsterdam Netherlands; ^10^ Department of Molecular Cellular Hemostasis Sanquin Research Amsterdam Netherlands; ^11^ Angelo Bianchi Bonomi Hemophilia and Thrombosis Center Fondazione IRCCS Ca’ Granda Ospedale Maggiore Policlinico Di Milano Milan Italy; ^12^ Department of Pathophysiology and Transplantation Università Degli Studi di Milano Milan Italy; ^13^ Division of Haematology Saint‐Luc University Hospital Catholic University of Louvain Brussels Belgium; ^14^ Institute of Social Sciences Ivo Pilar Zagreb Croatia; ^15^ Croatian Haemophilia Society Zagreb Croatia; ^16^ Division of Radiology IRCCS Ospedale Policlinico San Martino Genova Italy; ^17^ Department of Health Sciences (DISSAL) University of Genova Genova Italy; ^18^ Department of Coagulation Disorders Medical Clinic 2 University Hospital Frankfurt Frankfurt Germany; ^19^ Orthopedics and Traumatology Clinic IRCCS Policlinico San Matteo Foundation Pavia Italy; ^20^ Blaubeuren and Hemostasis Center South (Pediatric Practice) Institute For Pediatric Research and Further Education (IPFW) Blaubeuren Germany; ^21^ Faculty of Medicine Health and Social Care Canterbury Christ Church University Canterbury UK; ^22^ Coagulation Unit Department of Hematology Karolinska University Hospital Stockholm Sweden; ^23^ Hematology Department La Paz University Hospital Autonoma University Madrid Spain

**Keywords:** medicine, quality of life, interprofessional education, multidisciplinary approach, haemophilia, musculoskeletal ultrasound, nursing, intensive care medicine, early detection

## Abstract

The evolving haemophilia care landscape has evolved with personalised prophylaxis, new coagulation factor molecules, non‐replacement therapies, and gene therapy, leading to significantly reduced bleeding frequency and haemophilic arthropathy burden. With improved treatment outcomes, clinical priorities have shifted from bleed prevention to long‐term joint preservation and enhanced quality of life for people with haemophilia (PwH). Subclinical joint changes often go undetected without sensitive tools. To this end, point‐of‐care musculoskeletal ultrasound (POC MCK‐US) has emerged as a crucial way for early detection of joint abnormalities offering fast, non‐invasive, and repeatable assessments.  When embedded within a structured multidisciplinary team, POC MSK‐US facilitates real‐time, personalised decision‐making and enhances cross‐disciplinary communication. In addition, it provides valuable information to patients and caregivers about their joint health and prophylaxis success, improving patient adherence and self‐efficacy. Broader adoption of POC MSK‐US remains limited due to access to training, equipment, and standardised assessment frameworks. Promoting its implementation through interprofessional education, standardisation, and institutional support—particularly via societies like the European Association for Haemophilia and Allied Disorders (EAHAD)—will be key to expanding equitable access, improving patient outcomes, and ensuring comprehensive, proactive haemophilia management.

## 1 Modern Approach to Haemophilia Management

Recent advances in haemophilia management have dramatically shifted the therapeutic and diagnostic landscape. Personalised prophylaxis strategies with extended half‐life factor concentrates, non‐replacement therapies, and the emergence of gene therapy have led to significant reductions in bleeding episodes and severity of haemophilic arthropathy, redefining treatment goals and shifting the focus from preventing bleeds to preserving long‐term joint health and improving quality of life of people with haemophilia (PwH) [1].

Modern haemophilia treatment has moved beyond reactive management to a proactive, personalised care model (Figure 1). Prophylaxis has significantly reduced the frequency and severity of joint bleeding (haemarthrosis), and slowed the progression toward irreversible joint damage creating a wider therapeutic window for intervention at various stages of the haemophilic arthropathy cascade: recurrent joint bleeding, development of chronic hypertrophic synovial membrane and progression to osteochondral damage [2]. However, these alterations often remain subclinical and may go unnoticed by both PwH and the clinical team [3, 4, 5]. Therefore, it is essential to rely on objective sensitive diagnostic tools capable of identifying such changes at an early stage, in order to implement strategies or corrective measures aimed at halting or delaying the progression of joint damage [6, 7]. Accurate differential diagnosis in symptomatic joints with arthropathy is crucial as well, because joint bleeding, synovitis and osteochondral damage may each occur independently and do not correlate necessarily with physical examination and other traditional outcome measures (e.g. annual bleeding rate, pain and pharmacokinetics) [8]. In particular, the same prophylactic regimen can yield very different outcomes depending on how it is implemented in daily life.

**FIGURE 1:**
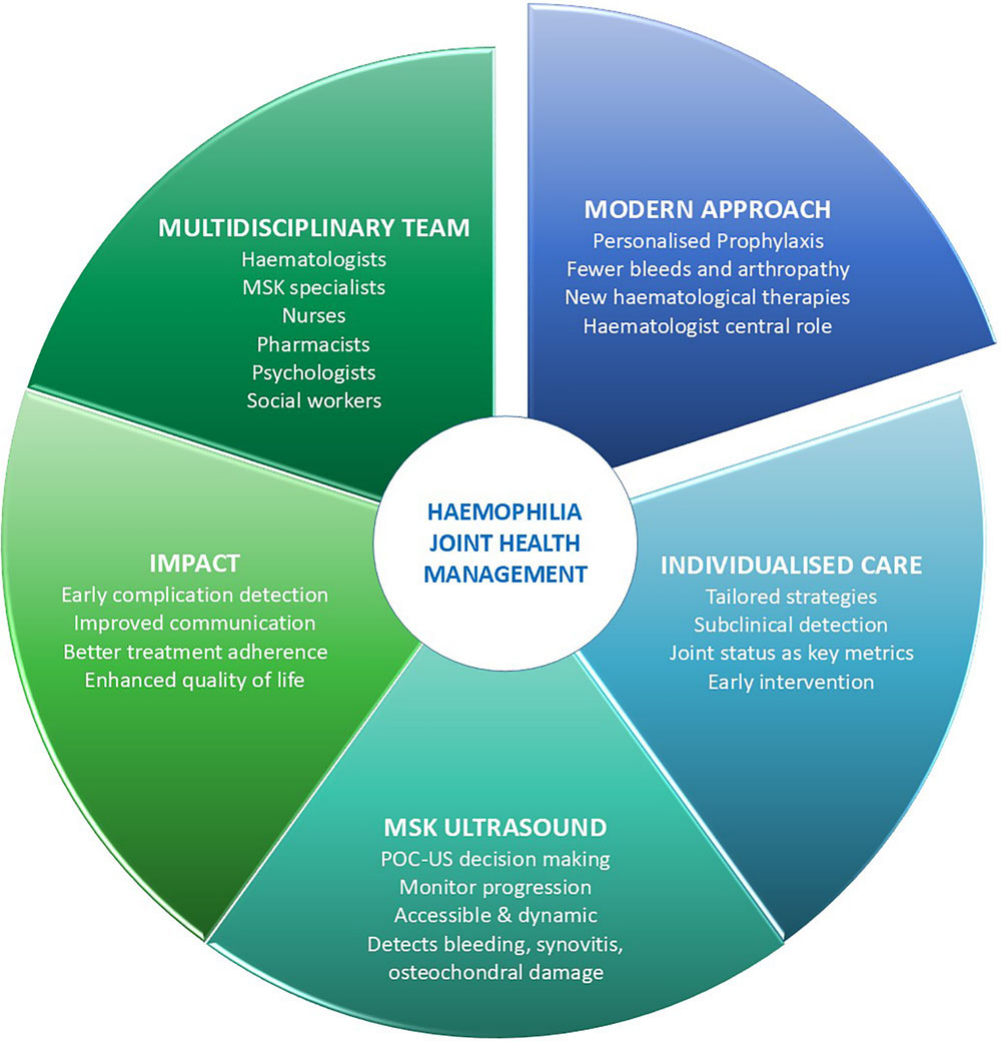
The main factors contributing to a modern approach to haemophilia management based on therapeutic advances and musculoskeletal ultrasound implementation. MSK: musculoskeletal; POC‐US: point‐of‐care ultrasound.

## 2 Individualised Care Through Objective Joint Assessment

Joint status is a key indicator in the haematological approach to haemophilia. A high number of confounding therapy‐related factors may go unnoticed during routine clinical visits, unless supported by objective assessment of joint status. To achieve a comprehensive evaluation of joint abnormalities and dysfunction, seamless communication and close, side‐by‐side collaboration among each professional are essential for accurate assessment and effective treatment in a structured multidisciplinary team including haematologists, physical and rehabilitation specialists, physiotherapists, rheumatologists, orthopaedic surgeons, psychologists, nurses and opened to imaging specialists (i.e. radiologists). Each contributes a unique perspective, and integration of these views ensures optimal treatment planning and monitoring (Table 1). Without structured communication pathways, however, critical clinical signals may be missed or misinterpreted, resulting in delayed interventions or inappropriate care.

**TABLE 1:** Usefulness of point of care musculoskeletal ultrasound information for the different members of the multidisciplinary team.

Healthcare professional	Usefulness of musculoskeletal ultrasound
Haematologist	It allows more accurate detection of joint status and progression of arthropathy, guiding decisions on haematological prophylaxis and facilitating appropriate planning of further procedures.
Musculoskeletal specialist^*^ and radiologists	It offers diagnosis of different intra‐articular problems, allowing more targeted application of exercise programmes, pain killers, intra‐articular procedures, orthoses, etc to improve pain and joint function safely.
Nurses	It provides valuable information for ongoing education in self‐care, self‐administration of prophylaxis, early recognition of signs of bleeding and adherence to treatment.
Psychologist	It helps to understand the challenges of chronic illness in order to better target mental health support, with the aim of improving adherence, emotional well‐being and community integration.
Social worker	It allows for a better understanding of the economic, social and employment challenges that may result from the physical problems patients live with, in order to implement coping strategies in the school or work environment and to manage care resources.
Patient and caregiver	It shows patients the status of their joints, which encourages adherence to prophylaxis and aids decision‐making about physical activity and prevention of complications. Visual education empowers the patient, allowing them to actively participate in the management of their disease.

* Specialists in physiatry, physiotherapy, rheumatology, occupational therapy, orthopaedic surgery, sports medicine, etc.

In this context, point‐of‐care musculoskeletal ultrasound (POC MSK‐US) provides valuable support in acute clinical situations by helping differentiate haemarthrosis from non‐bleeding causes of joint symptoms, thereby facilitating timely and appropriate management decisions. Serial ultrasound examinations may also assist in monitoring the resolution of bleeding episodes and guiding recovery and rehabilitation. These applications are particularly relevant because prompt recognition and optimal management of haemarthroses are essential to reduce the risk of persistent synovitis and subsequent joint deterioration. Moreover, some clinically symptomatic bleeding episodes may remain unrecognised as haemarthroses, highlighting the value of objective imaging assessment in routine haemophilia care.

## 3 POC MSK‐US as a Tool for Early Diagnosis and Monitoring

Published over a decade ago, POC MSK‐US scanning protocols have become increasingly used alongside personalised prophylaxis and new therapies, underscoring the need for sensitive, rapid, and accessible tools to monitor a disease that remains dynamic despite improved bleeding control [6]. Among these, the Haemophilia Early Arthropathy Detection with Ultrasound (HEAD‐US) protocol [9] has gained widespread use due to its simplicity, short examination time, and high sensitivity for early joint changes. The Joint Tissue Activity and Damage Examination (JADE) [10] represents a more comprehensive approach, and is particularly useful in research settings. POC MSK‐US has gained increasing prominence in clinical practice, due to the need for sensitive, rapid, and accessible tools to monitor a disease that remains dynamic despite improved personalised prophylaxis and novel therapies.

MSK‐US offers several distinct advantages: it is non‐invasive, low‐cost, free from ionising radiation, and well‐tolerated even in the paediatric population. When performed as a POC modality by trained non‐imaging professionals (primarily physical medicine and rehabilitation specialists, physiotherapists and rheumatologists) [11], it can provide timely, repeatable, and clinically meaningful insights that facilitate more immediate adjustments to treatment plans and enhance coordination among caregivers [12]. Detailed knowledge of joint status is relevant to the entire multidisciplinary team and should ideally be shared with the whole team to support joint decision‐making, particularly in complex cases.

## 4 Impact of Specific Joint Diagnoses on Clinical Decision‐Making

Major bleeds are usually visible and can be characterized on ultrasound, while minor and occult (subclinical) bleeds may not be directly detectable. Confirming joint bleeding enables prompt intervention, or in the cases of no bleeding, allows to avoid unnecessary interventions and enables targeted pain management, improving resource use.

Synovitis, detectable early via POC MSK‐US before clinical signs at a potentially reversible stage, serves as a key biomarker indicating that one or more bleeding episodes have occurred in that joint in the recent past, even if unnoticed by the patient, suggesting the need to adjust prophylaxis regimens and implement supportive measures to prevent recurrence even in the absence of overt bleeding [13, 14], and prompting measures such as radioisotopic or chemical synoviorthesis in refractory cases [15].

Early detection of osteochondral damage is crucial, as it allows for timely interventions that may slow joint deterioration and functional impairment, including compensatory changes in other joints, warranting more intensive rehabilitation even in patients on tertiary prophylaxis without active bleeding or detectable synovitis. Given the irreversibility of such changes, clinical management should prioritise pain control and functional support [15].

As joint status is dynamic and influenced by treatment adherence, physical activity, and comorbidities, longitudinal evaluation is essential, providing insight into treatment efficacy over time [12, 14, 16]. Regular scanning can identify occult disease, evolving pathologic changes and guide adjustments in management strategies [14], supporting a more personalised care across the entire team (Table 1). In particular, with HEAD‐US, POC MSK‐US has proven capable of providing responsiveness and sensitivity to change after treatment by detecting modifications in the volume of synovial thickening following the establishment of an appropriate therapy regimen [14].

## 5 The Multidisciplinary Team: Enhancing Coordinated Care and Patient Engagement

Knowledge of POC MSK‐US findings, such as the presence of synovitis or osteochondral damage, empowers the multidisciplinary haemophilia team to better coordinate care, tailor interventions, and optimise patient outcomes. (Table 1). For example, haematologists may identify those PwH who need better disease management and to act early on adherence and prophylactic treatments. Physiatrists can plan targeted pain management and mobility strategies, such as analgesia, ergonomics, orthoses and interventional procedures to optimise rehabilitation programmes, while physiotherapists can personalise and guide exercise regimens based on real‐time insights into joint status. Occupational therapists can recommend technical aids to prevent overuse in daily activities. Sports medicine professionals may adjust physical activity recommendations, fostering safe and active lifestyle personalizing physical activity recommendations. Orthopaedic surgeons may use US to complement other imaging techniques, refining indications and timing of surgical interventions, improving perioperative planning and minimizing complications. POC‐MSK‐US may also assist as a guide in mini‐invasive procedures such as intra‐articular injections, increasing accuracy and improving clinical outcomes [12, 16]. Nurses and psychologists made aware by the MSK specialists of POC MSK‐US findings can discuss with patients supporting self‐care, improving treatment adherence, enhancing patient engagement and reinforcing emotional support. Social workers can use joint assessment findings to plan individualised assistance, including technical aids, workplace accommodations, and home‐based support [12, 16]. As an educational tool for PwH, MSK‐US helps better understanding of their condition, empowering them to actively participate in shared decision‐making and supporting long‐term engagement with care plans.

Joint ultrasound allows patients to gain insight into the condition of their joints and helps detect possible joint bleeds—including those without clear or recognisable symptoms. This is particularly important in children, who may not be able to identify or articulate signs of bleeding, and who might sometimes intentionally conceal them in order to avoid infusions or the restrictions that come with acknowledging a bleed. As a non‐invasive, quick, and safe imaging method, ultrasound provides valuable feedback to patients on the appropriateness and effectiveness of their prophylactic treatment, encouraging patient adherence and thereby helping to prevent complications associated with undetected or untreated bleeding episodes.

The opportunity for patients to access ultrasound enables them to receive in a more timely manner a detailed analysis of musculoskeletal issues they might have, which then enables those issues to be treated as soon as possible. The reassurance this brings will add positively to a person's psychological wellbeing.

## 6 Future Directions and Implementation Challenges

The recent advent of ultraportable US devices is likely to significantly expand the reach and utility of POC MSK‐US for these professional figures. These compact, mobile systems enable more flexible and frequent assessments, promoting continuity of care, reducing diagnostic delays, and fostering a more proactive and patient‐centred approach to MSK health in haemophilia. Looking ahead, digital innovations such as telemedicine and artificial intelligence‐assisted image acquisition and detection of joint alterations may further expand equitable access to joint imaging with specialist input, training and remote supervision.

Multidisciplinary teams are essential for the comprehensive management of haemophilia; however, not all Haemophilia Centres are organised accordingly, often due to limited awareness of the value of team‐based care, as well as logistical and educational barriers. Inter‐societal initiatives aimed at assessing current practices across Haemophilia Centres will be strategic in identifying obstacles and implementing solutions to enhance cross‐disciplinary communication and patient outcomes.

Integrating POC MSK‐US into routine practice and strengthening interprofessional communication are key to optimising treatment and improving quality of life for PwH. As a shared language within the haemophilia team, MSK‐US serves not only as a diagnostic tool but also as a catalyst for coordinated care.

Despite its many advantages, POC MSK‐US remains underutilised in haemophilia management, primarily due to the limited number of trained professionals, restricted access to equipment and the time commitment required for both training and implementation [11]. To foster wider adoption, targeted efforts must focus on enhancing education and accessibility across disciplines. Standardisation of joint ultrasound terminology and assessment criteria through professional inter‐societal collaboration is essential to ensure consistent, structured communication regarding joint status and MSK health. This is critical both within multidisciplinary teams and across care centres to unlock the potential of POC MSK‐US in contemporary haemophilia care [17].

Structured training programs developed and offered at various competency levels (i.e. basic, intermediate, advanced), should be tailored specifically to each member of the multidisciplinary team. The establishment of accredited training Centres and mentorship networks within European or global haemophilia organisations, such as the European Association for Haemophilia and Allied Diseases (EAHAD) could facilitate capacity building and peer‐to‐peer learning. These programs should integrate theoretical knowledge with hands‐on practice, case‐based learning and a competency framework. Including POC MSK‐US modules into haematology fellowship curricula and healthcare professional training can promote early adoption and ensure a skilled multidisciplinary workforce.

Integrating POC MSK‐US into routine haemophilia care enhances diagnostic accuracy, supports timely and individualised decisions, improves communication and patient engagement; its implementation should be prioritised to optimise outcomes, quality of life and resource use through equitable access, training, and multidisciplinary collaboration.

## Funding

The authors have nothing to report.

## Conflicts of Interest

MTAR has participated as a speaker, in advisory boards and sponsored symposia, with Novo Nordisk, Takeda, Roche, Pfizer, Octapharma, Amgen, Novartis, CSL Behring and Sobi. MA payed Speaker/consultant: Sobi, Pfizer, Roche, Bayer, NovoNordisk and Baxter/shire. HDCR: reimbursements for symposia/congress attendance and/or speaking and/or consultancy fees, and/or research funding from Sobi, Roche, NovoNordisk, Pfizer, Takeda, Sanofi, Bayer, CSL Behring, Spark and Octapharma. KF has received unrestricted grants/research funding from CSL Behring, Sobi for research unrelated to the current study, consultancy fees from Sobi, Sanofi, Novo Nordisk and Roche (all fees to the institution). RG has participated in advisory boards of Bayer, Novo Nordisk, Roche, Sanofi, Sobi, speaker bureau/educational meetings for Novo Nordisk, Pfizer, Sobi, Kedrion. WM: Bayer, Biomarin, Biotest, CSL Behring, Chugai, Freeline, LFB, Novo Nordisk, Octapharma, Pfizer, Regeneron, Roche, Sanofi, sobi, Takeda/Shire, uniQure. DS received speaker honorarium from Sobi, Sanofi, and Roche. Research funding: Sobi, Novo Nordisk and Bayer. Educational grant: Sobi. GP: reimbursements for symposia/congress attendance and/or speaking and/or consultancy fees, and/or research funding from Bayer, Kedrion, NovoNordisk, Octapharma, Pfizer, Roche, Sobi, Takeda. All other authors declare no conflicts of interest. Other boards: KF, RG and CH are also part of the ISTH Standardization Subcommittee on Factor VIII, Factor XI, and Rare Coagulation Disorders, RG is member of the International Prophylaxis Study Group (IPSG) imaging expert working group.

## Data Availability

Data sharing not applicable to this article as no datasets were generated or analysed during the current study
